# Heritability of cephalometric variables of airway morphology in twins with completed active growth

**DOI:** 10.1186/s12903-023-02919-x

**Published:** 2023-04-27

**Authors:** Monika Šidlauskienė, Mantas Šidlauskas, Antanas Šidlauskas, Simonas Juzėnas, Kristina Lopatienė

**Affiliations:** 1 Department of Orthodontics, Faculty of Medicine, Lithuanian University of Health Sciences, Eivenių 2, Kaunas, LT-50161 Lithuania; 2 Institute of Digestive Research, Faculty of Medicine, Lithuanian University of Health Sciences, Eivenių 2, Kaunas, LT-50161 Lithuania; 3 Clinic of Orthodontics, Medical Academy, Lithuanian University of Health Sciences, J. Lukšos-Daumanto str. 6, Kaunas, LT-50106 Lithuania

**Keywords:** Twin study, Upper airway, Cephalometrics, Genetics, Orthodontics

## Abstract

**Background:**

The interplay between genetic and environmental impacts on dental and facial morphology has been widely analyzed, but little is known about their relative contributions to airway morphology. The aim of this study was to evaluate the genetic and environmental influences on the cephalometric variables of airway morphology in a group of postpubertal twins with completed craniofacial growth.

**Materials and methods:**

The materials comprised lateral head cephalograms of 94 pairs of twins (50 monozygotic, 44 dizygotic) with completed craniofacial growth. Zygosity was determined using 15 specific DNA markers. The computerized cephalometric analysis included 22 craniofacial, hyoideal, pharyngeal structural linear and angular variables. Genetic analysis and heritability estimation were performed using maximum likelihood genetic structural equation modeling (GSEM). Principal component analysis (PCA) was used to assess the correlations between cephalometric measurement variables.

**Results:**

Upper airway dimensions showed moderate to high genetic determination (SPPW-SPP and U-MPW: a^2^ = 0.64 and 0.5, respectively). Lower airway parameters showed only common and specific environmental determination (PPW-TPP a^2^ = 0.24, e^2^ = 0.38; LPW-V c^2^ = 0.2, e^2^ = 0.63; PCV-AH c^2^ = 0.47, e^2^ = 0.28). The relationship between the maxilla and the hyoid bone (for variables PNS-AH, ANS-AH d^2^ = 0.9, 0.92, respectively) showed very strong additive genetic determination. The size of the soft palate was affected by additive and dominant genes. Its length (SPL) was strongly influenced by dominant genes, while its width (SPW) showed a moderate additive genetic influence. Owing to correlations in the behavior of variables, the data could be expressed in 5 principal components that jointly explained 36.8% of the total variance.

**Conclusions:**

The dimensions of the upper airway are strongly determined by genes, while the parameters of the lower airway depend mainly on environmental factors.

**Trial registration:**

The protocol has been approved by the Kaunas Regional Ethical Committee (No. BE – 2–41., May 13, 2020).

## Introduction

The airway, mode of breathing, and craniofacial formation are very closely interrelated during growth and development [1]. It is known that dysfunction of the human airway and breathing can cause malocclusion and skeletal deformation. [2]. An open bite, a hyperdivergent growth pattern, proclined upper incisors, increased lower facial height, steepening of the mandibular plane angle, lowering of the chin and increase in the gonial angle are among these features [3, 4].

Nasal breathing abnormalities may develop due to a variety of conditions, such as adenoid and tonsil hypertrophy, mandibular or maxillary retrognathism, a short mandibular body, and backward and downward rotation of the mandible, which may lead to upper airway stenosis, reduction of the pharyngeal airway space and even the development of obstructive sleep apnea (OSA) [5, 6]. Obesity increases any present airway obstruction by enlarging the tongue, uvula and throat tissues [7, 8]. All of these conditions, as well as facial phenotype and dental and skeletal morphology, are influenced by genes and the environment. The interplay between genetic and environmental impacts on dental and facial morphology has been widely analyzed, but little is known about their relative contributions to airway morphology [9–11].

The prognosis of the success for orthodontic and dentofacial orthopaedic correction of malocclusion is determined by the extent to which a particular malocclusion can be influenced by therapeutic environmental intervention. Generally, malocclusions with a genetic cause are thought to be less amenable to treatment than those with an environmental cause. The same is truth for the success of airway morphology improvement by means of corrective orthodontics and orthopaedics [12]. Therefore, knowledge of genetic and environmental impact on airway structures, is of primary interest for orthodontic research and clinical practice [13].

Although the use of comprehensive phenotype analysis in combination with large-scale genome-wide association studies maximizes the efficiency with which clinically relevant phenotype–genotype correlations can be detected, only a few correlations of this type have been discovered. Significant genetic contributions to variables such as the timing of dental maturation, incisor and canine crown diameters, missing or supernumerary teeth, arch dimensions and Class III malocclusion development have been established [14]. However, data concerning genetic and environmental influences on airway morphology are scarce and mainly related to sleep apnea cases [15, 16]. Determining the degree of influence exerted by genetics and by environmental factors, such as orthodontic treatment, in the development of airway obstruction can help shed light on the role of orthodontists in addressing this health issue.

Twin studies combined with advanced statistical methods provide an opportunity to determine the relative contributions of genetics and environment to dentofacial development [10, 11, 14].

The aim of this study was to evaluate the genetic and environmental influences on the cephalometric variables of airway morphology in a group of postpubertal twins with completed craniofacial growth.

## Materials and methods

The study was undertaken in the Department of Orthodontics, Lithuanian University of Health Sciences (LSMU). The sample consisted of 94 pairs of same-gender twins (50 monozygotic, 44 dizygotic) selected from the register of the Twin Centre at LSMU. The protocol was approved by Kaunas Regional Ethical Committee (No. BE – 2–41). All twins had clinical consultations, and lateral cephalograms necessary for this study were performed. The CVM method was used to assess the completion of skeletal maturation [17].

Inclusion criteria: twins of European origin, cervical vertebral maturation (CVM) stage 6 (active growth completed), high-quality cephalometric data available from both twins in the database.

Exclusion criteria: previous orthodontic treatment, permanent tooth extractions, dental or facial trauma, systemic diseases or syndroms.

### Zygosity determination

All participating twins underwent DNA tests to determine their zygosity [18].

Zygosity determination was carried out using a DNA test. The polymerase chain reaction set AmpFLSTR Identifiler (Applied Biosystems, USA) was used to amplify short tandem repeats, and 15 specific DNA markers (D8S1179, D21S11, D7S820, CSF1PO, D3S1358, TH01, D13S317, D16S539, D2S1338, D19S433, vWA, TROX, D18S51, D5S818, FGA) and the Amel fragment of the amelogenin gene were used for comparison of genetic profiles. Zygosity determination using this molecular genetic technique has 99.9% accuracy [18, 19].

### Cephalometric analysis

The cephalometric analysis was used to measure airway and skeletal dimensions. The cephalograms were taken in centric occlusion under standard conditions using digital X-ray equipment. For standardized positioning, a cephalostat was used to stabilize the subject’s head in a constant position relative to the sensor. Lateral cephalometric (LC) radiographs were taken after swallowing. All lateral cephalograms had the same magnification. The radiographs were analyzed by using Dolphin Imaging v.11.7.

**Fig. 1:**
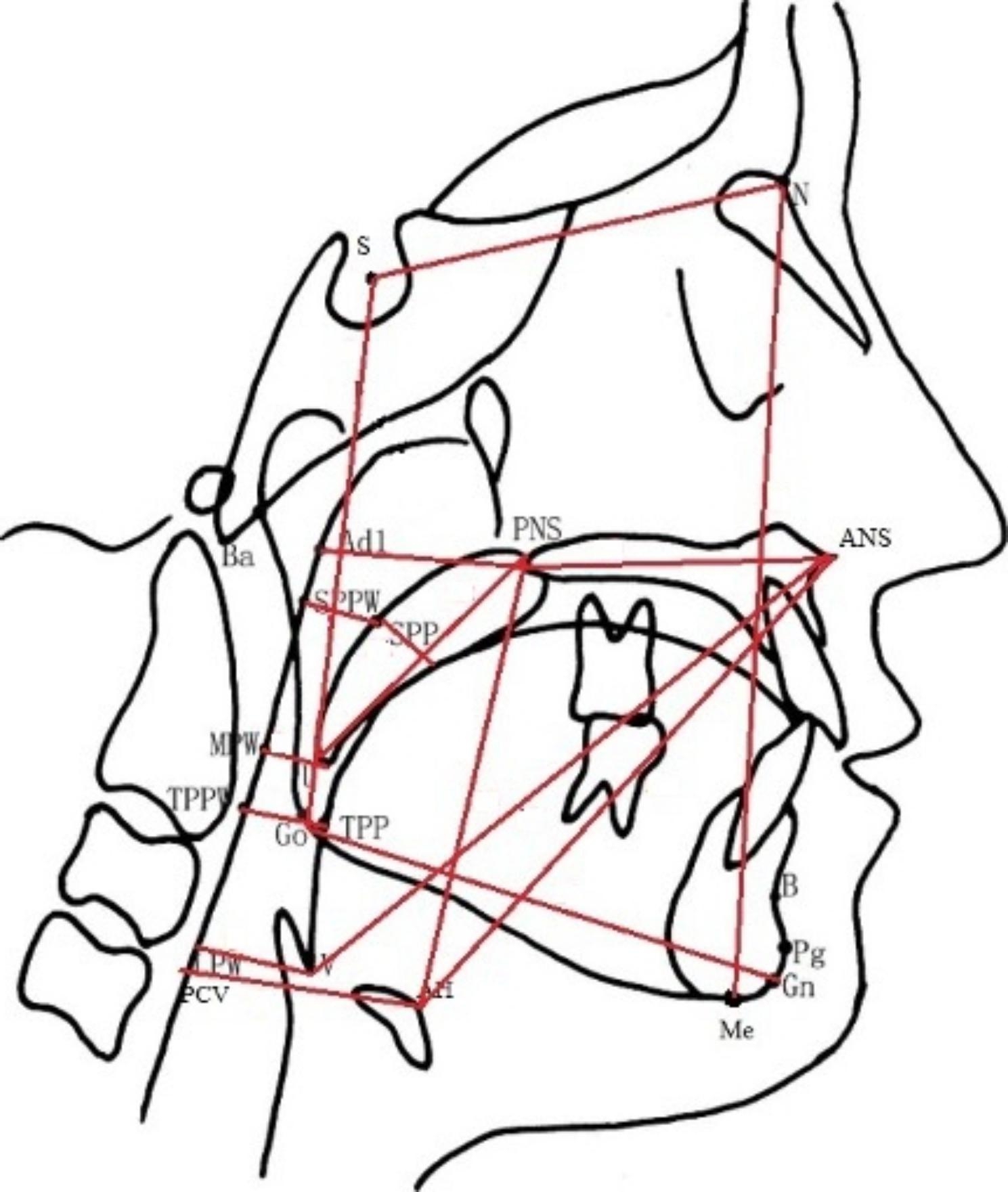
Definitions of cephalometric landmarks used in the study

Definitions of cephalometric landmarks, reference lines, and cephalometric measurements are presented in Fig. 1.

Cephalometric points: *S, sella –* the midpoint of the hypophyseal fossa; *N, nasion –* the anterior point at the frontonasal suture; *A, point A –* the deepest point in the curvature of the maxillary alveolar process; *B, point B –* the deepest point in the curvature of the mandibular alveolar process; *ANS, point ANS –* the anteriormost point of the anterior nasal spine; *PNS, point PNS –* the posteriormost point of the hard palate; *Ad1, point Ad1 –* the point of intersection of the posterior pharyngeal wall and line PNS-Ba; *SPPW, point SPPW –* the point of intersection of the posterior pharyngeal wall and the line that extends perpendicularly from the posterior pharyngeal wall to the center of the soft palate; *SPP, point SPP –* the point of intersection of the posterior margin of the soft palate and the line that extends perpendicularly from the posterior pharyngeal wall to the center of the soft palate; *MPW, point MPW –* the middle pharyngeal wall, located at the intersection of the posterior pharyngeal wall and the line extending perpendicularly from that surface to U; *TPPW, point TPPW –* the point of intersection of the posterior pharyngeal wall and the extension of line B-Go; *LPW, point LPW* – the point on the posterior pharyngeal wall from which a perpendicular line will pass through point V; *PCV, point PCV –* the point of intersection of the posterior pharyngeal wall and an extension of the lower edge of the second cervical vertebra; *U – uvula*, tip of the uvula; *V, vallecula –* the point where the epiglottis meets the base of the tongue; *AH, anterior hyoid –* the most anterior and superior point on the body of the hyoid bone, representing the inferior part of tongue; *Gn, gnathion –* the midpoint between Pogonion and Menton; *Go, gonion –* the mid-plane point at the gonial located by bisecting the posterior border lines of the mandible; *Me, menton –* the lowest mandible anterior point.

Cephalometric variables: *PNS-Ad1* – distance between PNS and Ad1; *SPPW-SPP* – distance between SPPW and SPP; *U-MPW* – distance between U and MPW; *PPW-TPP* – distance between PPW and TPP; *LPW-V* – distance between LPW and V; *PCV-AH* – distance between PCV and AH; *S-N* – distance between S and N; *N-Me* – distance between N and Me; *S-Go* – distance between S and Go; *PNS-ANS* – distance between PNS and ANS; *SPL* – soft palate length; *SPW* – soft palate width; *PNS-AH* – distance between PNS and AH; *ANS-AH* – distance between ANS and AH; *ANS-V* – distance between ANS and V; *Go-Gn* – distance between Go and Gn; *Ulip-E* – distance between upper lip anterior border and E line; *Llip-E* – distance between lower lip anterior border and E line; *Wits* – distance perpendicular to points A and B onto the occlusal plane in mm; *SNA* – angle determined by points S, N and A; *SNB* – angle determined by points S, N, B; *SN-MP* – angle formed by Go-Me.

### Method error

Intraobserver method error was checked on 20 randomly selected patients’ cephalograms with the method offered by Bland and Altman [20]. Cephalograms were traced twice after a one-month interval.

### Estimation of heritability

Genetic structural equation modeling (GSEM) was performed using the “OpenX” package [21]. Classical univariate ACE and ADE twin models were fitted to the gender-adjusted cephalometric measurement data. The models were used to estimate the significance of the different components of total phenotypic variance (P), which is equal to the sum of the following variance components: the additive genetic factor (A), the shared environment (C), the nonadditive genetic factor (D), and the unique environment (E). The goodness of fit of the complete and reduced ACE and ADE models relative to a perfectly fitted (saturated) model was measured by the Akaike information criterion (AIC) [22]. The model of each cephalometric variable with the lowest AIC value was selected as the best fitting model.

### Principal component analysis

Principal component analysis (PCA) was used to reduce the dimensionality of cephalometric measurement data and to check the correlations between variables. PCA was performed using the “principal” function from the “psych” package (Procedures for Psychological, Psychometric and Personality Research: https://cran.r-project.org/web/packages/nFactors/index.html). The principal components were rotated using varimax rotation. The number of components was determined using the “nScree()” function from the “nFactors” package according to the optimal coordinates index. A variable belonged to a component if the absolute value of the component loading was larger than 0.5.

### Statistical analysis

Statistical analyses were performed in the statistical computing environment R (version 3.3.0). P values below 0.05 were considered statistically significant.

## Results

### Method error

The results of error analysis found no significant differences between the initial and repeated measurements (Table 1).

**Table 1:** Method error determined by a Bland–Altman plot for repeatability of the cephalometric measurements, with statistical significance calculations

Variable	SE	p
PNS-Ad1	0.24	N.S.
SPPW-SPP	0.43	N.S.
U-MPW	0.30	N.S.
PPW-TPP	0.23	N.S.
LPW-V	0.48	N.S.
PCV-AH	0.41	N.S.
S-N	0.50	N.S.
N-Me	0.81	N.S.
S-Go	0.65	N.S.
PNS-ANS	0.47	N.S.
SPW	0.60	N.S.
SPL	0.21	N.S.
PNS-AH	0.22	N.S.
ANS-AH	1.15	N.S.
ANS-V	1.16	N.S.
Go-Gn	0.28	N.S.
SNA	0.41	N.S.
SNB	0.26	N.S.
ANB	0.45	N.S.
SN-MP	0.65	N.S.
Ulip-E	0.35	N.S.
Llip-E	0.16	N.S.
WITs	0.09	N.S.

SE – error of method, expressed as standard error; p – probability that the means of the first and second measurements differed as assessed by the Wilcoxon signed-rank test; NS – not significant

### Estimation of heritability

The AIC was calculated for each parameter, and the AIC values of each model were analyzed. Only the lowest values were chosen and considered to be the most suitable model for further analysis. The contribution of factors (a^2^, c^2^, d^2^, e^2^) of the best-fitting model for each parameter was counted. The results of the model-fitting analysis are presented in Tables 2 and 3.

Variables representing upper airway dimensions (SPPW-SPP, U-MPW) showed moderate to high genetic determination (AE model), with a^2^ = 0.64 and 0.5, respectively; PNS-Ad1 had strong dominant determination (DE), with d^2^ = 0.51. Lower airway parameters were mostly determined by environmental factors. PPW-TPP, LPW-V, and PCV-AH showed only common and specific environmental dependency.

Skeletal variables were all dependent on genetics to some extent. Maxilla length and had high dominant genetic determination, and Go-Gn and S-Go showed additive genetic, common environmental, and specific environmental influences. N-Me length was affected by additive genetic factors and by common and specific environmental influences.

The size of the soft palate was determined by additive and dominant genetic factors. Its length (SPL) was strongly influenced by dominant genetic factors, while its width (SPW) showed a moderate additive genetic influence.

Variables reflecting the relationship between the maxilla and the hyoid bone (PNS-AH, ANS-AH) showed very strong additive genetic determination, with d^2^ = 0.9 and 0.92, respectively.

The parameters representing the sagittal position of the mandible and its relationship with the cranial base and lip position were all strongly influenced by genetics. Angles SNA and SNB fit best to the model determined by additive genes and specific environment. Angle SN-MP was determined by specific and common environmental factors, angle Ulip-E was determined by dominant genetic factors, and Llip-E was determined by additive genetic factors.

**Table 2:** AIC values of all the models

	ACE	ADE	DE	AE	CE	E
PNS-Ad1	2.48	-1.02	**-3.02**	0.48	10.93	18.99
SPPW-SPP	3.40	3.72	4.74	1.71	4.69	29.32
U-MPW	-5.66	-5.65	-5.83	**-7.64**	-1.88	24.46
PPW-TPP	4.09	4.29	3.30	**2.29**	2.81	15.77
LPW-V	14.57	16.02	15.03	14.02	**12.56**	14.07
PCV-AH	3.71	8.60	11.73	6.60	**1.82**	44.24
S-N	-0.36	-2.16	**-3.90**	-2.36	25.66	54.44
N-Me	**-7.86**	-1.45	6.41	-3.45	9.48	114.47
S-Go	**2.04**	12.45	21.17	10.49	8.41	115.43
PNS-ANS	-7.19	-7.76	**-9.71**	-9.19	-4.96	3.55
SPW	-10.47	-10.21	-10.65	**-12.21**	-11.55	0.82
SPL	6.84	1.93	**-0.07**	4.84	40.90	64.09
PNS-AH	-8.52	-8.50	-6.41	**-10.5**	33.87	95.01
ANS-AH	-7.51	-7.52	-6.3	**-9.51**	47.11	113.63
ANS-V	-3.02	-2.94	0.2	**-4.94**	28.8	81.16
Go-Gn	**-4.29**	-0.68	3.26	-2.68	-1.08	70.22
SNA	-2.89	-2.89	-3.2	**-4.78**	28.49	94.44
SNB	-2.77	-2.87	-3.66	**-4.77**	28.49	94.44
ANB	3.36	2.44	1.44	**1.35**	32.2	64.97
SN-MP	-0.49	4.54	6.86	2.54	**-2.5**	33.25
Ulip-E	-9.25	9.72	-10.93	**-11.25**	12.09	53.27
Llip-E	-3.00	-4.28	**-6.19**	-5.00	21.44	65.40
WITs	2.52	-1.25	**-3.25**	0.52	22.21	39.98

E – specific environmental factors; CE – common and specific environmental factors; AE – additive genetic factors and specific environmental factors; ACE – additive genetic factors, common environmental factors, and specific environmental factors; ADE – additive genetic factors, dominant genetic factors, and specific environment; DE – dominant genetic factors and specific environmental factors; values in **bold** – best-fitting models (lowest AIC values)

**Table 3:** Best-fitting models for each variable

	a^2^	SE (a^2^)	d^2^	SE (d^2^)	c^2^	SE (c^2^)	e^2^	SE (e^2^)
PNS-Ad1 (DE)			0.51	0.08			0.19	0.08
SPPW-SPP (AE)	0.64	0.08					0.24	0.08
U-MPW (AE)	0.50	0.08					0.22	0.07
PPW-TPP (AE)	0.24	0.09					0.38	0.09
LPW-V (CE)					0.20	0.10	0.63	0.10
PCV-AH (CE)					0.47	0.06	0.28	0.06
S-N (DE)			0.77	0.04			0.09	0.04
N-Me (ACE)	0.21	0.02			0.14	0.14	0.05	0.02
S-Go (ACE)	0.89	0.13			0.3	0.12	0.07	0.02
PNS-ANS (DE)			0.48	0.08			0.21	0.08
SPW (AE)	0.46	0.08					0.24	0.08
SPL (DE)			0.81	0.03			0.08	0.03
PNS-AH (AE)	0.9	0.02					0.4	0.02
ANS-AH (AE)	0.92	0.01					0.03	0.01
ANS-V (AE)	0.86	0.02					0.06	0.02
Go-Gn (ACE)	0.05	0.2					0.23	0.04
SNA (AE)	0.78	0.03					0.09	0.03
SNB (AE)	0.84	0.02					0.07	0.02
ANB (AE)	0.8	0.03					0.08	0.03
SN-MP (CE)					0.42	0.69	0.3	0.07
Ulip-E (AE)	0.75	0.04					0.1	0.04
Llip-E (DE)			0.76	0.04			0.1	0.04
WITs (DE)			0.7	0.05			0.12	0.05

a^2^ – additive genetic factors; d^2^ – dominant genetic factors; c^2^ – common environmental factors; e^2^ – specific environmental factors; SE – standard error.

### Principal components

According to the correlations in the behavior of the variables, the data were reduced to 5 principal components, which jointly explained 36.8% of the total variance (Table 4). The first component (PC1) showed correlations with the Go-Gn, LPW-V, N-Me, PCV-AH, PNS-ANS, S-Go, S-N, and SPW and explained 23.5% of the total variance. This component represented linear variables describing dimensions of the face and was highly influenced by genetics. The second principal component (PC2) showed strong correlations with angles PNS-Ad1, PPW-TPP, SPPW-SPP, and U-MPW, which explained 13.2% % of total variance and showed high genetic determination. The third component (PC3) showed correlation of 3 variables ANB, Ulip-E, Llip-E that represent lips position and sagittal jaw position relationship. PC4 showed correlation with SN-MP, SNA, SNB and this component describes jaws relationship with cranial base. PC5 showed correlation ANS-AH, ANS-V, PNS-AH, SPL.

**Table 4:** Factor loadings after varimax rotation

	PC1	PC2	PC3	PC4	PC5
ANB	-0.13	0.19	**0.71**	0.01	0.12
ANS_AH	0.25	0.11	0.13	-0.12	**0.85**
ANS_V	0.34	0.08	0.14	-0.11	**0.76**
Go_Gn	**0.72**	0.13	-0.24	0.14	0.14
Llip_E	0.03	0.01	**0.77**	-0.15	-0.10
LPW_V	**0.51**	0.24	0.01	0.14	0.03
N_Me	**0.67**	-0.06	-0.01	-0.46	0.32
PCV_AH	**0.71**	0.12	-0.09	0.10	0.11
PNS_Ad1	0.01	**0.62**	-0.10	0.30	0.19
PNS_AH	0.37	-0.08	0.05	-0.01	**0.76**
PNS_ANS	**0.61**	-0.04	0.10	0.03	0.18
PPW_TPP	0.29	**0.61**	0.09	-0.01	-0.35
S_Go	**0.58**	-0.05	-0.19	0.35	0.41
S_N	**0.53**	0.14	-0.15	0.01	0.28
SN_MP	0.03	-0.23	0.17	**-0.78**	-0.09
SNA	0.26	0.08	0.33	**0.80**	-0.06
SNB	0.35	-0.06	-0.07	**0.83**	-0.10
SPL	0.17	-0.18	-0.09	0.13	**0.6**
SPPW_SPP	-0.11	**0.85**	-0.01	0.07	0.05
SPW	**0.65**	-0.13	-0.01	0.11	0.22
U_MPW	0.22	**0.83**	0.08	-0.05	-0.15
Ulip_E	-0.10	-0.06	**0.88**	-0.05	-0.04
WITs	-0.04	-0.06	0.39	0.13	0.12

Values in bold: factor loadings that are significant at p > 0.05

## Discussion

Understand upper airway morphology, assessing its heritability and knowing characteristics of its growth in a healthy population could help doctors identify persons at risk of breathing problems, such as snoring, OSA or mouth breathing, and even improve the treatments available to patients [23–25].

Heritability was analyzed to understand how upper airway morphology was influenced by genetic factors. The results of our study showed that 19 of 23 cephalometric parameters were strongly determined by genetics, while the remaining parameters were strongly influenced by environmental factors or both genetic and environmental factors. The considerable influence of genetic factors on pharyngeal space variations has been studied by Billing et al. [26]. The study participants were 19 monozygotic and 23 dizygotic twin pairs. The results of that study showed that the size of the pharyngeal space, the thickness of the posterior nasopharyngeal wall and the nasopharyngeal airway are strongly influenced by genetic factors. J. H. Kang et al. measured pharyngeal parameters using lateral cephalograms of adult monozygotic and dizygotic twins. They also found that airway structures were under strong genetic control [27].

These findings are in agreement with the results of our study: the nasopharyngeal airway measurement (PNS-Ad1) was influenced by genetic factors (a^2^ = 0.51). This might be explained by the fact that the nasopharyngeal area is surrounded by the body of the sphenoid bone, the basilar part of the occipital bone and the arch of the atlas on the posterior and superior sides; the morphology of these bony structures are strongly determined by genetic predisposition. On the other hand, the nasopharynx communicates with the oropharynx on the inferior side and the soft palate on the superior side, and these airways are necessary for speech, breathing and swallowing [28]. This could explain the weak environmental determination of the linear parameter PNS-Ad1 (e^2^ = 0.19).

There is research showing that obesity is also related to reduced upper airway dimensions [9]. Although the environment plays a role in the development of obesity, body mass index (BMI) is correlated within families, but never the less, twin studies demonstrate an important role of genetics in the development of obesity [29].

The oropharyngeal airway space (U-MPW) was determined by additive genetic factors (a^2^ = 0.5). The high heritability of this trait means that the oropharyngeal airway space is strongly influenced by genetic factors. This is in contrast to the results of previous studies, which have suggested that the oropharynx is more likely to be related to environmental factors, such as posture, than to genetic factors and that surrounding soft tissues are more influenced by environmental factors [30].

The oropharynx has an important role in orthodontic treatment planning. It has been reported that rapid maxillary expansion (RME) causes not only an increase in dental width but also changes in the oropharyngeal airway space [31]. After orthodontic treatment with the RME/Hyrax appliance, the volume of the oropharyngeal airway increased, and the results persisted in the long term after controlling for growth. Other investigations showed that the oropharyngeal airway volume did not change after orthodontic treatment with RME compared to that of the control group [32]. These contradictory results may be due to the use of different methods, an insufficient sample size or inaccuracies in measurement. Orthodontic treatment with fixed orthodontic appliances and the use of functional appliances such as the Herbst appliance increase airway volume and reduce resistance to airflow [33–36]. However, the oropharyngeal airway space (U-MPW) was also affected by environmental influences (e^2^ = 0.22), which, although statistically nonsignificant in the overall sample, can also be crucial for some individuals. This might be because the oropharynx is surrounded by the tongue and the hyoid bone on the anterior side and the cervical vertebrae on the posterior side; these structures can change their positions [37].

The upper airway space has been studied by orthodontists for its close relation to the jaws and the craniofacial morphology. Some studies have revealed that the respiratory system is related not only to upper airway size but also to malocclusion type or craniofacial structures [38]. In the present study, we did not find any significant correlation between the sagittal spatial relationships of skeletal structures and the upper airway dimensions. This correlation is still controversial among researchers. Di Carlo G et al. reported that there are no significant relationships between the sagittal jaw structure and the upper airway volume [39].

Our results showed that hypopharyngeal structures are under environmental influence. It is known that there is a direct correlation between pharyngeal space and obesity [40]. According to Andrew M. Kim et al., tongue volume and tongue fat are increased in patients with OSA. These researchers claim that fat deposition not only influences tongue size but may also decrease tongue force and hinder the tongue from properly functioning as an upper airway dilator muscle. These findings coincide with those of our study, which showed that hypopharynx dimensions are affected by environmental factors [41].

Contrary to the environmental influence hypothesis, J. H. Kang showed that the structure of the hypopharynx has high heritability. This contradiction of our findings and the findings of J. H. Kang et al. could be due to inaccuracy of measurement because the vallecula can collect saliva, preventing initiation of the swallowing reflex. These measurements can also be influenced by head posture, cervical spine position and craniofacial angulation. Da Costa et al. stated that exact measurements of hyoid bone position through cephalometric analysis are difficult because even small deviations may generate apparent variation in the location of the hyoid [42].

Some of the limitations that we encountered in this study are common for research of this nature. The most common limitation in twin studies is sample size [43]. It is well known that twin births account for only a small proportion of births; for example, the twin birth rate in Lithuania was 11.7 per 1,000 births (Medical Data of Births 2014). In the present study, participants were required to meet certain conditions. Additionally, participation in this research was voluntary, which also reduced the sample size of twins.

Since most studies use two-dimensional cephalometry for orthodontic diagnosis and treatment planning, it is not surprising that some difficulties are encountered. The main problems that orthodontists face are difficulties in evaluating three-dimensional structures of the upper respiratory tract with two-dimensional cephalometric analysis, difficulties in identifying the landmarks, and overprojection [18, 44]. The hyoid triangle method, despite being used as a standard method for assessing hyoid bone position in lateral cephalometric images, is not applicable to 3D image analysis [45]. In comparison with cone-beam computed tomography (CBCT), lateral cephalometric (LC) imaging is a preferable tool to measure linear and angular parameters and is a valuable instrument in the screening process [46]. Despite certain limitations, studies with twins are informative and a useful method to evaluate genetic and environmental influences on phenotype [47]. The findings from the present study could help orthodontists, otolaryngologists, speech-language pathologists and pediatricians better understand what role heredity and environment plays in airway width. These findings might also be useful for diagnosing and planning treatment. Further research using CBCT or MRI and investigating larger sample sizes would be relevant and helpful.

## Conclusions

The dimensions of the upper airway are strongly determined by genes, while the parameters of the lower airway are mainly affected by environmental factors.

## Data Availability

The datasets used and/or analyzed during the current study are available from the corresponding author on reasonable request.

## Abbreviations

CVM: cervical vertebral maturation
CBCT: cone-beam computed tomography
DNA: deoxyribonucleic acid
GSEM: genetic structural equation modeling
LC: lateral cephalometric (imaging)
LSMU: Lithuanian University of Health Sciences
OSA: obstructive sleep apnea
RME: rapid maxillary expansion
